# Functional independence, frailty and perceived quality of life in patients who developed delirium during ICU stay: a prospective cohort study

**DOI:** 10.1186/s40001-023-01530-8

**Published:** 2023-12-04

**Authors:** Beatriz Lobo-Valbuena, Rosario Molina, Raúl Castañeda-Vozmediano, Leire Lopez de la Oliva Calvo, Ana Abella, María-Mercedes Garcia-Arias, Irene Salinas Gabiña, Federico Gordo

**Affiliations:** 1 Intensivist – Intensive Care Unit, Hospital Universitario del Henares, Coslada, Madrid Spain; 2 Grupo de Investigación en Patología Crítica, Facultad de Ciencias de La Salud, Universidad Francisco de Vitoria, Pozuelo de Alarcón, Madrid Spain; 3 Research Support Unit, Faculty of Medicine, Universidad Francisco de Vitoria, Pozuelo de Alarcón, Madrid Spain

**Keywords:** Activities of daily living, Critical care, Delirium, Health-related quality of life, Post-intensive care syndrome

## Abstract

**Background:**

Survivors of critical illness are frequently left with a long-lasting disability. We hypothesised that patients who developed delirium during ICU stay, compared with patients who did not, would have worse health-related quality of life following a critical illness.

**Methods:**

Prospective longitudinal observational and analytical study assessing functional independence, frailty and perceived quality of life measured with the Barthel Index, the Clinical Frailty Scale, and the SF-36, comparing patients who developed delirium during ICU stay and patients who did not. The questionnaires were used at different times during the follow-up (upon ICU admission, at ICU discharge, at hospital discharge and 2 years after hospital discharge).

**Results:**

In a cohort of 1462 patients, we matched 93 patients who developed delirium (delirium group) with 93 patients who did not develop delirium (no-delirium group). Of 156 completed questionnaires (84.7%), we observed that (a) in each of the two groups of patients, the scores related to functional independence (Barthel Index) and frailty (Clinical Frailty Scale) tended to improve over time (*p* < 0.001), being consistently less favourable in the delirium group compared to the no-delirium group (*p* < 0.001); (b) the patients who developed delirium also presented lower scores on the SF-36 scale, these differences being statistically significant, and therefore evidencing a worse quality of life, with impact on both the psychological and social spheres (*p* < 0.001).

**Conclusions:**

Patients who developed delirium had significantly lower scores 2 years after hospital discharge on the three used questionnaires, displaying a clear negative impact on the physical, psychological, and social dimensions. The study's results reinforce the need to support and strengthen the care of ICU survivors.

**Supplementary Information:**

The online version contains supplementary material available at 10.1186/s40001-023-01530-8.

## Background

Delirium is a severe neuropsychiatric disorder of organic origin characterised by the appearance of alterations in both consciousness and cognitive function [1]. The number of patients admitted to the intensive care unit (ICU) is increasing, and more than 80% survive ICU care [1, 2]. Subsequently, increasing attention is being given to the long-term outcomes of ICU survivors.

Clinical guidelines [3] have recommended using a bundle approach (e.g. ABCDEF bundle) to target eliminating multiple modifiable risk factors of ICU (delirium is in a percentage preventable) [3–5], reducing the chances of suffering delirium or shortening its duration once established. The development of delirium during the ICU stay carries a worse short-term prognosis, such as increased mortality [2], longer duration of mechanical ventilation, longer ICU length-of-stay, and cognitive impairment [6–8]. Along with this, delirium is also associated with poor long-term prognoses, such as persistent cognitive impairment [9–11] and disability in activities of daily living, including worse motor-sensory function [9–13].

The medical and technological advances made in intensive care have allowed us to treat pathologies that, decades ago, presented an ill-fated prognosis. The professional expertise in critical illness has expanded beyond that required for essential illness management and organ support, including understanding the risk factors for and consequences of critical illness [14]. In this situation, and while Intensive Care Medicine physicians and other healthcare professionals involved in critical care have always been aware of the difficulties involved in recovery after discharge from ICU admission, the literature addressing the devastating long-term consequences of the illness on critically ill patients and their family members [15, 16] is recent. Collectively defined as post-intensive care syndrome (PICS), it has been estimated to occur in 30–50% of patients. PICS involves a new or worsening physical and psychological health status alteration, which appears and persists after hospitalisation for a critical illness. Risk factors associated with developing PICS include delirium during admission, immobility, deep sedation, systemic corticosteroids, and prolonged mechanical ventilation [17, 18].

Thus, improving our understanding of the risk factors amenable to intervention and their implication for patient prognosis (both short and long-term) may lead to improvements in clinical care and foster the development of post-ICU care programmes [19–22]. Thus, the Society of Critical Care Medicine released the ABCDEF bundle, which encourages assessment and management of pain, agitation, and delirium, implementation of early mobilisation, and family engagement, along with a recommended strategy to mitigate the risk of PICS [3, 20].

We hypothesise that patients who developed delirium during ICU stay suffered more difficulties in subsequent recovery after discharge from the hospital, negatively impacting the patient's functional independence and perceived quality of life. We aimed to study functional independence, frailty and quality of life in a two-year follow-up in two cohorts of patients, the difference between them being whether or not they developed delirium during their ICU stay.

## Methods

We conducted a longitudinal observational and analytical study including prospectively collected data from a cohort of patients admitted to a general ICU from October 1, 2016, up to May 1, 2019. Data were collected prospectively in the Registry of the University Hospital of Henares Intensive Care Unit. The research was approved by the Francisco de Vitoria University's Healthcare Ethics Committee (44/2018). Participation and acceptance of inclusion of patient’s data into the Registry were obtained by signing the informed consent document (by the patient or by an authorised surrogate in case the patient could not express their opinion). The study includes all patients admitted during the mentioned period who agreed to participate in the Registry. Exclusion criteria were patients under 18 years old, patients who died during follow-up and patients who required transfer to another hospital (given the impossibility of correct data collection and follow-up upon discharge).

We collected relevant demographic and clinical data from every patient, using the Simplified Acute Physiology Score (SAPS-3) on admission as a validated score for the severity of illness [23]. Delirium screening using the CAM-ICU (Confusion Assessment Method for the Intensive Care Unit) was performed by our nursing staff every eight hours (once every shift). In case of doubt, it was discussed with the attending physician. To be diagnosed with delirium, the patient needed to have a RASS [Richmond Agitation-Sedation Scale, [24]] score above -3 and a positive CAM-ICU (defined as an acute change or fluctuation in mental status, accompanied by inattention and either disorganised thinking or an altered level of consciousness [1, 25–27]). Before the study, training in the use of CAM-ICU was provided to the nursing staff. The delirium assessment rate of trained nurses was, therefore, high.

We located patients who had not developed delirium with similar demographic characteristics to those with delirium. Matching was performed using four variables: sex, age (± 3 years), the reason for admission (postoperative, cardiological, respiratory, neurological, sepsis, miscellaneous) and SAPS-3 (± 3). When matching, the first two criteria (same sex and age) were met. The third criterion to be completed was the reason for admission, and the fourth was the SAPS-3.

**Fig. 1:**
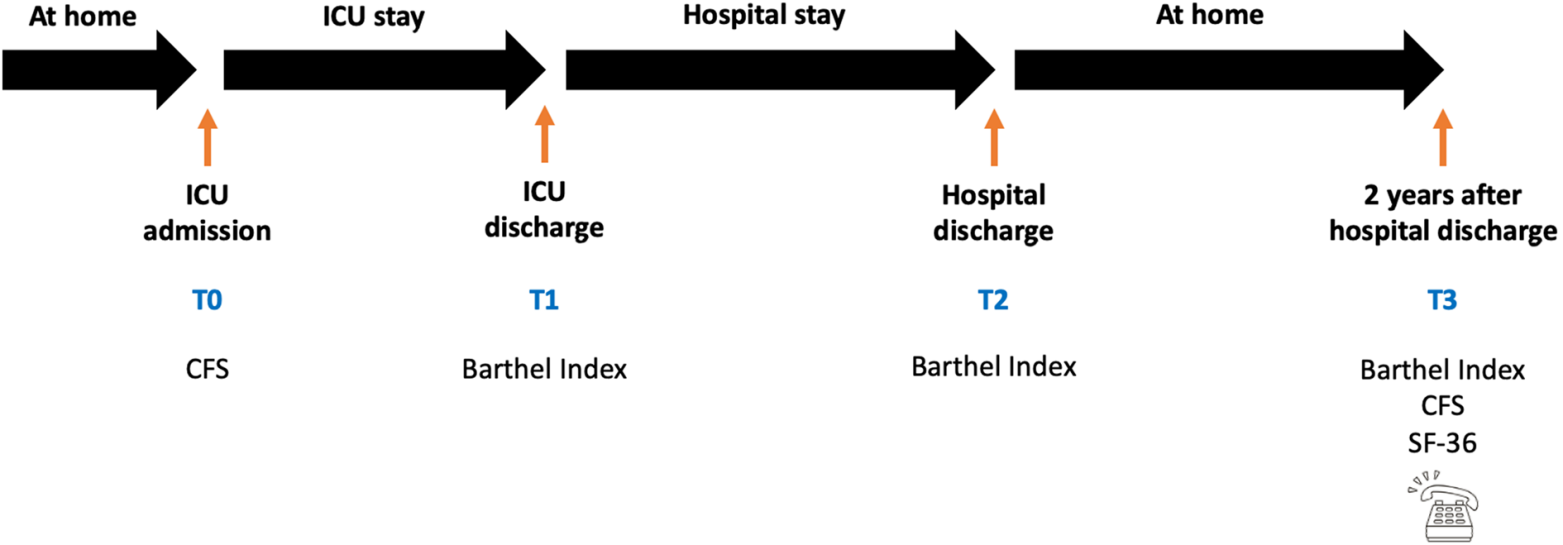
Flowchart of performed questionnaires during the study

Regarding the analysis of functional independence, frailty and quality of life, we used the following tools: Barthel Index (BI; a generic measure of physical or functional disability, which assesses the patient's level of independence concerning basic activities of daily living [28]), Clinical Frailty Scale (CFS; assessment of frailty [29–33]) and the 36-Item Short Form Health Survey (SF-36; composed of 36 questions (items) that assess both positive and negative states of health [34, 35]). We were able to collect BI at ICU discharge (referred to as T1), at hospital discharge (referred to as T2), and 2 years after hospital discharge (referred to as T3); CFS collected upon ICU admission (referred to as T0) and CFS at T3; and SF-36 at T3. Assessment of functional independence, frailty and perceived quality of life 2 years after discharge was carried out by telephone (Fig. 1).

### Statistical analysis

Categorical variables are expressed as numbers and percentages, while quantitative variables are expressed as medians and interquartile ranges. Assumptions of normality (assessment of normal distribution using the Anderson–Darling test) were checked.

Two ART-ANOVA (Aligned Ranks Transformation ANOVA) models were used to analyse the CFS and BI scores, with data obtained at different times (T0, T1, T2, T3) in two defined groups (patients who developed delirium and patients who did not develop delirium during ICU stay). Post hoc multiple comparisons of the main and interaction effects studied were performed with Bonferroni adjustment using the Signs test and Chi-square test, respectively [36]. For the study of simple effects, the Signs test was used to assess the impact derived from the difference between groups at each time individually and between times for each group separately, as the assumptions of normality and symmetry could not be assured. SF-36 results (obtained only at T3) were analysed using the Wilcoxon signed-rank test with continuity correction. The effect size, and thus the magnitude or intensity of the differences found, was assessed using Eta-squared (*η*^*2*^) for the main and interaction effects and *VD. A* (Vargha and Delaney's A) [37, 38] for the simple effects.

Statistical analysis was conducted using SPSS 20.0 software for Windows (IBM, Inc.; Illinois, USA) and R version 3.6.3 (R Core Team, 2020). P values < 0.05 were considered statistically significant, and 95% confidence intervals were evaluated for each estimate.

## Results

During the study period, 1534 patients were admitted to our ICU. Seventy-two patients were excluded from the statistical analysis (due to loss of data related to hospital transfer), obtaining a cohort of 1462 adult patients. The demographic and clinical characteristics of the 1462 patients, including short-term outcomes and multivariate analysis, are described elsewhere [39].

Ninety-three patients developed delirium during ICU stay (incidence of 6.3%), paired with 93 patients who did not develop delirium, totalling 186 patients. The demographic, clinical characteristics and response rate to the telephone survey are shown in Table 1.

**Table 1:** Demographics and clinical characteristics of the studied population

		*N* = 186	No delirium *N* = 93	Delirium *N* = 93
Age, yr, median [IQR]		70 [61–80, 8]	70 [61–80]	71 [60–81]
Sex	Male, *n* (%)	114 (61.3%)	56 (60.2%)	58 (62.4%)
	Female, *n* (%)	72 (38.7%)	37 (39.8%)	35 (37.6%)
Main diagnosis on admission, n (%)	Acute respiratory failure	44 (23.7%)	22 (23.7%)	22 (23.7%)
	Postoperative	50 (26.9%)	25 (26.9%)	25 (26.9%)
	Sepsis	38 (20.4%)	19 (20.4%)	19 (20.4%)
	Acute cardiac disease (*)	20 (10.8%)	10 (10.8%)	10 (10.8%)
	Coma	18 (9.7%)	9 (9.7%)	9 (9.7%)
	Cardiac arrest	8 (4.3%)	4 (4.3%)	4 (4.3%)
	Shock other than sepsis	8 (4.3%)	4 (4.3%)	4 (4.3%)
Comorbidities, n (%)	Cardiovascular	120 (64.5%)	61 (65.6%)	59 (63.4%)
	Respiratory	57 (30.6%)	30 (32.3%)	27 (29.0%)
	Renal	28 (15.1%)	11 (11.8%)	17 (18.3%)
	Hepatic	29 (15.6%)	14 (15.1%)	15 (16.1%)
	Cancer disease	66 (35.5%)	40 (43.0%)	26 (28.0%)
	Endocrine	84 (45.2%)	39 (41.9%)	45 (48.4%)
SAPS-3, median [IQR]		57.5 [4–66]	56 [48–64]	59 [49–66]
Number of organ failure/s, median (IQR)		2 [1–3]	2 [0–2]	3 [2–4]
Organ-supportive treatments	Invasive MV, *n* (%)	88 (47.3%)	30 (32.3%)	58 (62.4%)
	Days under invasive MV, days (IQR)	4 [2–10]	2 [1–5]	6.5 [3–15]
	Reintubation, n (%)	7 (3.8%)	2 (2.2%)	5 (5.4%)
	Non-invasive MV, *n* (%)	24 (12.9%)	14 (15.1%)	10 (10.8%)
	CRRT, *n* (%)	8 (4.3%)	3 (3.2%)	5 (5.4%)
LOS ICU, days, median (IQR)		4 [2–9]	3 [2–5]	7 [3–15]
LOS hospital after ICU discharge, days, median (IQR)		8 [4–16]	8 [4–15]	10 [5–18]
Unplanned readmission to ICU, *n* (%)		8 (4.3%)	1 (1.1%)	7 (7.5%)
Telephone survey response	No, *n* (%)	30 (16.1%)	8 (8.6%)	22 (23.7%)
	Yes, *n* (%)	156 (83.9%)	85 (91.4%)	71 (76.3%)
Telephone survey recipient	Patient, *n* (%)	146 (93.6%)	81 (95.3%)	65 (91.5%)
	Surrogate, n (%)	10 (6,4%)	4 (4.7%)	6 (8.5%)

*Yr*  years, *IQR*  interquartile range

(*) Acute coronary syndrome, acute heart failure, rhythm disorders

Five patients who developed delirium died during the study period. Two years after hospital discharge, we obtained 156 responses (84% response rate). These 156 patients completed the required information for the BI, the CFS, and the SF-36 physical data (85 patients in the non-delirium group and 71 patients in the delirium group). Ten surrogates provided answers to the telephone survey due to language problems (in 4 patients) and old age with associated cognitive impairment (in 6 patients). These ten responses were excluded when analysing the rest of the SF-36 items (we considered that the surrogate answers were not valid when assessing the psychological impact on the individual patients), making a total of 146 responses (81 patients in the non-delirium group and 65 patients in the delirium group).

Overall scores obtained in both groups for BI and CFS are shown in Table 2, and the complete statistical analysis can be found in the Supplement material. Both scores were always significantly different (T1, T2 and T3), except for CFS obtained in T0 (upon ICU admission). Likewise, the effect size was of moderate-–large magnitude for all taken times.

**Table 2:** Barthel Index and CFS scores

Survey	Time	No delirium	Delirium	*p* (*)	*VD.A* (**)
Barthel index, median, [IQR]	ICU discharge (T1)	70 [47, 5–100]	50 [30–65]	< 0.001	0.707^b^
	Hospital discharge (T2)	90 [55–100]	55 [50–85]	< 0.001	0.713^a^
	2 years after hospital discharge (T3)	100 [90–100]	85 [75–98, 8]	< 0.001	0.679^b^
*Clinical Frailty Scale*, median, [IQR]	On ICU admission (T0)	3 [3–4]	3 [3–4]	0.361	0.457
	Two years after hospital discharge (T3)	3 [3–4]	4 [3–4, 8]	< 0.001	0.307^b^

IQR = interquartile range; *VD.A* = Vargha and Delaney’s A

(*) = Signs test sign test for related samples

(**) = Regarding size effect with *VD.A*

^a^Large effect (≥ 0.71 or ≤ 0.29)

^b^Moderate effect (0.64–0.71 < or > 0.29–0.34)

^c^Small effect (between 0.56–0.64 or 0.34–0.44). Values close to 1 or 0 indicate a strong intensity of the mean difference

**Fig. 2:**
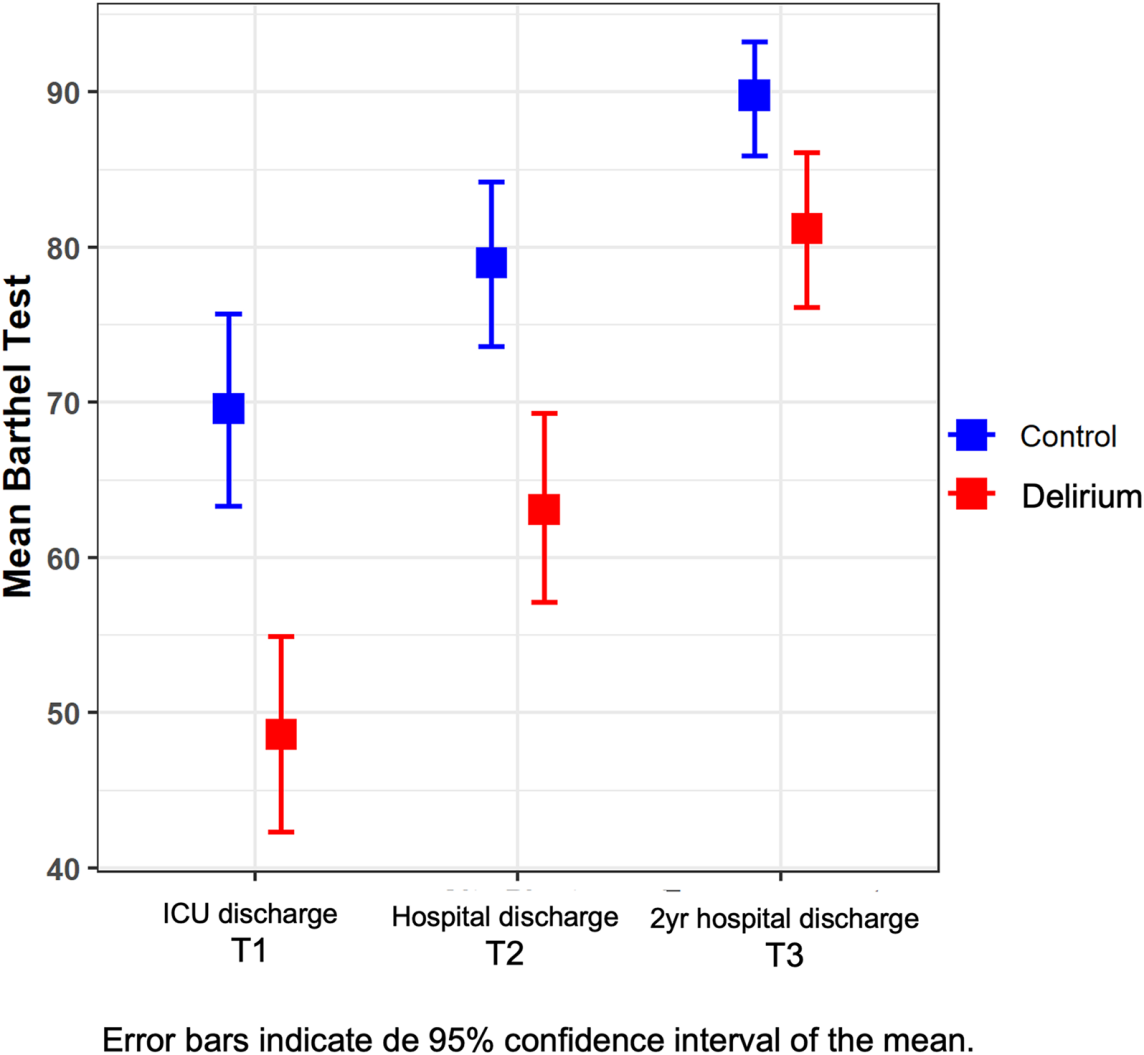
Time course of barthel index

**Fig. 3:**
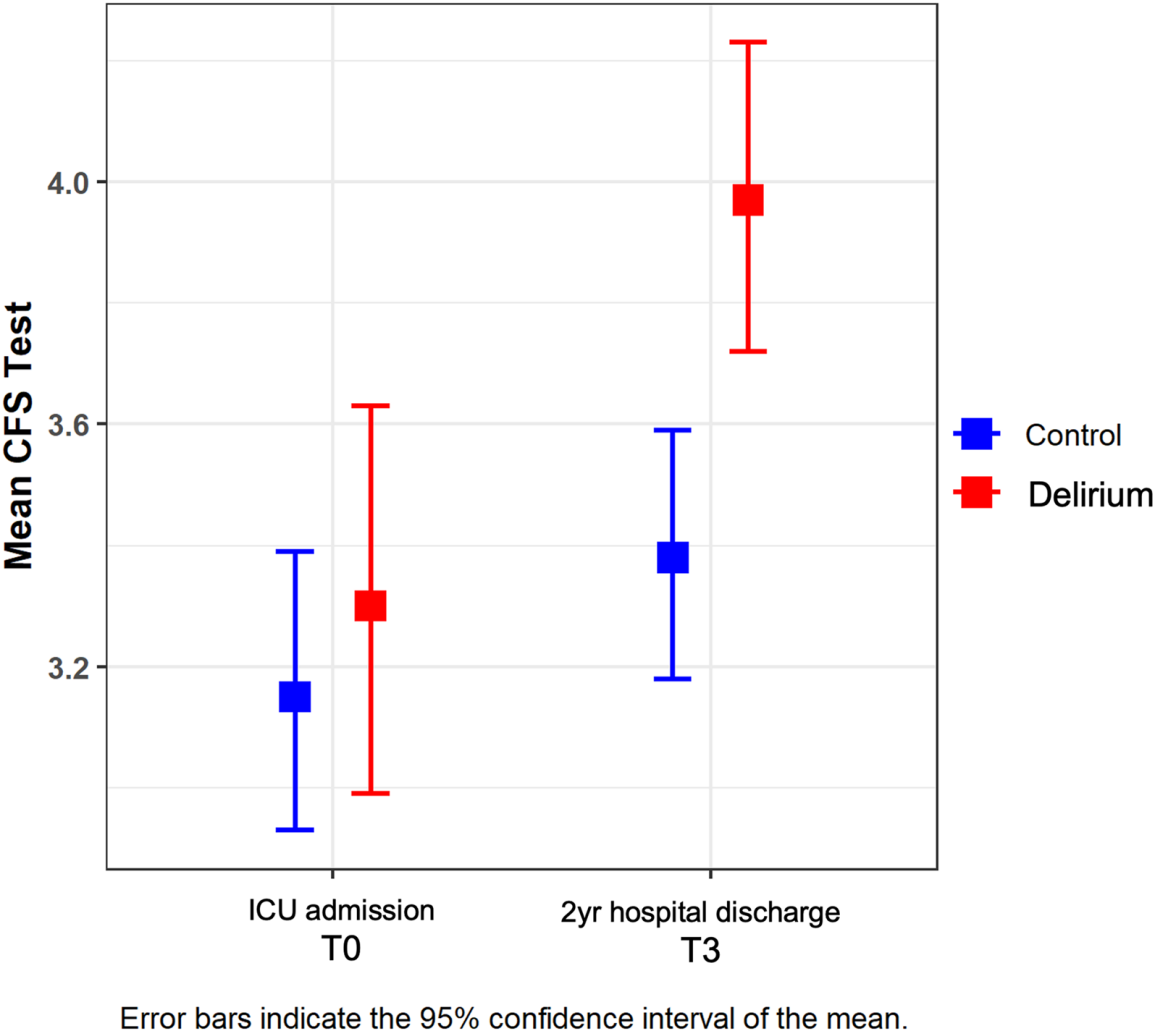
Time course of clinical frailty scale

We also studied the time pattern of the BI and CFS scores, plotted in Figs. 2, 3. Concerning the BI (Fig. 2), patients who did not develop delirium (in blue; no-delirium group) obtained significantly better scores than patients who did develop delirium (in red; delirium group) (*F* = 69,134, *p* < 0.001), although the difference between the two groups of patients did not remain constant over time (*F* = 8,897; *p* < 0.001) (Additional file 1: Table S1). In particular, the differences between groups are significantly smaller at T3 (Additional file 1: Table S2) compared to those found on T1 (*p* = 0.011) and T2 (*p* < 0.001), although at all three times the differences between groups are significant (Table 2). Moreover, significant differences were found between the three times for both groups individually (*p* < 0.001) (Additional file 1: Table S3). Regarding the CFS (Fig. 3), scores collected on ICU admission (T0) did not differ between the two groups. Notwithstanding, a statistically significant difference was observed at T3 (Table 2), with a lower CFS value (and therefore a better baseline situation) for the patients who had not developed delirium (*p* < 0.001) being that difference between groups significantly greater at T3 than at T0 (*F* = 9101; *p* = 0.003). Moreover, significant differences were found between the two times for the delirium group (*p* < 0.001) but not for the no-delirium group (*p* = 0.541) (Additional file 1: Table S3). The observed effect size (measured with η2; Supplementary Material) was more significant for the BI than the CFS (with a *η*^2^ > 0.14—high effect size—when assessing the impact of both defined main effects—delirium and time—on the BI) (Additional file 1: Table S1).

Results obtained using the SF-36 questionnaire 2 years after hospital discharge are shown in Table 3. Patients who developed delirium during their ICU admission had lower scores than those who did not develop delirium. Moreover, these differences were statistically significant for the nine items assessed (thus displaying a clear negative impact on the physical, psychological, and social dimensions). We also observed a moderate–large effect size, measured by the *VD.A* statistic for all SF-36 items.

**Table 3:** SF-36 questionnaire scores

Survey	SF-36 item	No delirium	Delirium	*p* (*)	*VD.A* (**)
SF-36 performed two years after hospital discharge, median, [IQR]	Limitations of activities	67.5 [35–85]	40 [2, 15–66]	< 0.001	0.707 ^b^
	Physical health problems	75 [25–100]	25 [0–75]	0.002	0.68 ^b^
	Emotional health problems	100 [7–100]	33.3 [0–100]	0.005	0.656 ^b^
	Energy	50 [30–70]	35 [15–50]	0.004	0.689 ^b^
	Mental health	56 [40–72]	36 [28–60]	0.001	0.705 ^b^
	Social activities	75 [5, 50–87]	50 [25–75]	0.004	0.68 ^b^
	Pain	77.5 [65–100]	67.5 [5, 45–77]	0.03	0.639 ^b^
	General health	45 [35–50]	40 [25–45]	< 0.001	0.713 ^a^
	One-year health transition	50 [30–50]	25 [25–50]	< 0.001	0.307 ^b^

*IQR* interquartile range, *VD.A*  Vargha and Delaney’s

(*) = Wilcoxon signed-rank test for related samples

(**) = Regarding size effect with VD.A:

^a^Large effect (≥ 0.71 or ≤ 0.29)

^b^Moderate effect (0.64–0.71 < or > 0.29–0.34)

^c^Small effect (between 0.56–0.64 or 0.34–0.44). Values close to 1 or 0 indicate a strong intensity of the mean difference

## Discussion

This prospective cohort study examined the plausible relationship between patients’ long-term prognosis and the development of delirium during ICU stay. A statistically significant negative impact, of moderate magnitude, was observed during the 2-year up. Patients with delirium present a significantly increased risk of poor functional independence, frailty and quality of life, measured using the Barthel Index, Clinical Frailty Scale and SF-36.

ICU-acquired delirium is associated with adverse short- and long-term outcomes. In the long term, it is strongly related to functional impairment in activities of daily living, as well as cognitive deficits, memory deficits, and inattention [10, 40]. Compared to the general population, ICU survivors have a lower health-related quality of life one year after critical illness [41] and a higher prevalence of long-term cognitive impairment [42]. Although other studies [8, 13] have not found such a strong association between the development of delirium and long-term quality of life, they have demonstrated its association as an independent risk factor for self-reported long-term cognitive functioning problems (measured with the EQ-6D scale or European Quality of Life—six dimensions self-classifier).

Using the BI for activities of daily living enables the assessment of functional outcomes of patients. Li et al. [43] assessed frailty and the development of delirium in cardiac surgery patients. They observed that the coexistence of preoperative frailty and postoperative delirium development led to a substantial loss of independence in three to four basic activities of daily living and a 30.2-fold increased probability of dying one year after surgery. Besides, frail patients who developed delirium also scored lower on the different quality-of-life used questionnaires [44–46]. Regarding CFS upon admission, we observed no statistically significant differences between the two groups, probably related to the fact that, at that time, the patients had not yet developed delirium.

The use of SF-36 to assess the impact of delirium on the patient's quality of life is more limited. The assessment of the psychological dimension is understandably tricky, given that many other factors (and not only those related to a residual effect of previous ICU admission) may affect the psyche of patients. A prospective study [40], with a follow-up of 18 months, assessed the impact of delirium on cognitive function and long-term health-related quality of life. No differences were found in the overall assessment of the SF-36 between survivors with and without delirium. However, those survivors who had experienced delirium reported significantly more social errors, and their total score on the cognitive failure questionnaire was considerably higher. Another study [47], focusing on using the SF-36 as a predictor of postoperative delirium, found that some SF-36 scores (especially the general health perception score) were significantly lower in patients with postoperative delirium. Regarding our study, we found a significant impact in all the items assessed by SF-36, highlighting a solid association (large effect size) on the negative impact over the item "one-year health-transition". It seems clear that the development of delirium affects a patient's physical performance and quality of life, including their mental health and social life (encompassing aspects such as the patient's relationship with others or their return to the workforce).

Our study has several potential limitations. First, we performed a single-centre analysis with a relatively limited number of patients. However, we believe that the single-centre nature of the study may favour the homogeneity of data and management consistency. Secondly, matching was performed using four demographic variables and not more, given the limited number of patients within the study sample. Thirdly, the selection of a follow-up period two years after discharge from the hospital could have also led to a bias, as it fails to accurately account for whether the patient developed episodes of delirium post-ICU discharge, post-ICU rehabilitation, access, transfer to rehabilitation facilities or need of specific ambulatory care other than scheduled routine check-ups. Fourthly, we have faced the challenges of conducting long-term outcome studies in critical care [48, 49]. As more patients survive a critical illness, attention has shifted to assessing long-term morbidity and quality of life. However, this increased interest has shed light on many important methodological challenges in conducting this research. Significant challenges have involved retaining patients in the follow-up study, reducing threats to internal and external validity, and—as far as possible—achieving sufficient statistical power when matching (we were able to pair one patient who did not develop delirium with one patient who did, with an estimated statistical power of 60%). Regarding the loss of patient follow-up, 30 patients (16.1%) did not respond to the telephone surveys, in addition to another ten patients who could not adequately complete the entire content of the questionnaires. The social science literature suggests a minimum rate of 70–80% [50], which was exceeded in our work, although no such guidelines exist for ICU survival studies. Strategies described to mitigate this problem include using a patient contact system or scheduling and close follow-up of the cohort [51, 52]. Fifth, we found difficulty in obtaining homogeneous results regarding questionnaire responses. We could only perform a more exhaustive analysis of the time effect (main effect of time) for the BI, as we obtained results at the three previously defined times (T1, T2 and T3). By the same token, the SF-36 survey was only used two years after hospital discharge, precluding a temporal analysis of the results. Sixth and finally, the telephone survey collection process for a group of patients included in the study occurred during the COVID-19 pandemic. For this reason, although the data are presented as ‘‘surveys collected 2 years after hospital discharge’’, some patients did not answer the surveys until 2.5 to 3 years later. Moreover, we must highlight that we did not assess our patients' cognitive impairment adequately. We considered cognitive assessment during the study design process [11, 53–56] but finally discarded it, given that an adequate evaluation of this requires a healthcare visit. This could have harmed the number of patients who completed the follow-up. Besides, if we had decided to include this cognitive assessment and scheduled the hospital visits, the study could not have been carried out due to the COVID-19 pandemic and the mobility restriction measures put in place [57].

The present study also has several strengths. Considering our relatively small ICU capacity (between 8 and 10 available ICU beds), we included a high sample size. We applied a reasonably new statistical model, which allowed us to assess survey scores in two groups, measured at different times. The findings of our study reinforce our commitment to continue working on our multidisciplinary protocol for the management of post-ICU syndrome (coordinating both the hospital team and the Primary Care health centres attached to the hospital area to which we belong). Our post-discharge follow-up programme ensures a successful handover with the inpatient ward team and discusses the next steps with the patient and family. We also offer a support programme for relatives and caregivers, considering the patient’s values and wishes in the shared decision-making process. Despite being a young programme, so far, we have achieved encouraging results, observing improvement in components of mental health (fear, self-esteem, coping, sleep disorders), in the patient's ability to perform basic activities of daily living and in the perceived caregiver overload [58–60].

## Conclusions

On the three questionnaires, we observed significantly lower scores two years after hospital discharge (Barthel Index, Clinical Frailty Scale and SF-36). This difference was found to be maintained over time for the Barthel Index and the CFS. Our data suggest that patients who developed delirium had worse functional independence, frailty and perceived quality of life than patients who did not.

### Supplementary Information

**Additional file 1: Table S1.** Analysis of main effects and interaction effect for Barthel and CFS. **Table S2.** Post-hoc analysis for the Barthel Index. **Table S3.** Simple effects when studying the difference between the times for both groups.

## Data Availability

All data generated or analysed during this study are included in this published article.

## Abbreviations

ART-ANOVA: Aligned ranks transformation-analysis of the variance
BI: Barthel Index
CAM-ICU: Confusion assessment method for the ICU
CFS: Clinical frailty scale
EQ-6D: European quality of life—six dimensions self-classifier
ICU: Intensive care unit
IQR: Interquartile range
PICS: Post-intensive care syndrome
RASS: Richmond agitation-sedation scale
SAPS-3: Simplified acute physiology score-3
SARS-CoV-2: Severe acute respiratory syndrome coronavirus type 2
SF-36: 36-Item short form health survey
T0: Defined time or moment at ICU admission
T1: Defined time or moment at ICU discharge
T2: Defined time or moment at hospital discharge
T3: Defined time or moment 2 years after hospital discharge
*VD.A*: Vargha and Delaney's A
Yr: Years
*η*^2^: Eta-squared
