# Supplementary material for: Functional independence, frailty and perceived quality of life in patients who developed delirium during ICU stay: a prospective cohort study
Source: Eur J Med Res. 2023 Dec 4;28:560. doi: 10.1186/s40001-023-01530-8 (PMC10696684; doi:10.1186/s40001-023-01530-8)
Supplement: Supplementary file 1 — Additional file 1: Table S1. Analysis of main effects and interaction effect for Barthel and CFS. Table S2. Post-hoc analysis for the Barthel Index. Table S3. Simple effects when studying the difference between the times for both groups. [file 40001_2023_1530_MOESM1_ESM.docx]

**Additional file Material: ART-ANOVA statistical results**

**Table S1**. Analysis of main effects and interaction effect for Barthel and CFS

| **Survey** | **Studied effect** | ***F*** | ***gl*** | ***p*-value *** | ***η^2^* **** |
| --- | --- | --- | --- | --- | --- |
| **Barthel** | Main effect delirium | 69,134 | 1 | <0,001 | 0,146 ^a^ |
|  | Main effect time | 75,402 | 2 | <0,001 | 0,285 ^a^ |
|  | Interaction effect delirium - time | 8,397 | 2 | <0,001 | 0,043 ^b^ |
| **CFS** | Main effect delirium | 10,320 | 1 | 0,002 | 0,041 ^b^ |
|  | Main effect time | 30,553 | 1 | <0,001 | 0,120 ^c^ |
|  | Interaction effect delirium - time | 9,101 | 1 | 0,003 | 0,039 ^b^ |

*F* = ART ANOVA statistics. *gl*= degrees of freedom. *η^2^* = eta-squared

* Statistically significant difference was observed in all sections, showing lower scores in the delirium group versus de non-delirium group for BI and CFS.

** Regarding size effect with *η*^2^: ^a^ large effect (*η*^2^> 0,14); ^b^ moderate effect (*η*^2^ ≈ 0,06); ^c^ small effect (*η*^2^ ≈ 0,01)

**Table S2**. Post-hoc analysis for the Barthel Index

|  | ***Post-hoc*** | **Statistics** | ***p*** |
| --- | --- | --- | --- |
| Main effect time | T1 - T2 | 79** | <0,001 |
|  | T1 - T3 | 21** | <0,001 |
|  | T2 - T3 | 17** | <0,001 |
| Interaction effect between *delirium* and time | *Delirium* and (T1 - T2) | 1,544*** | 0,642 |
|  | *Delirium* and (T2 - T3) | 7,621*** | 0,011 |
|  | *Delirium* and (T1 - T3) | 16,026*** | <0,001 |

** = Signs test with Bonferroni adjustment; *** = Chi squared with Bonferroni adjustment

**Table S3**. Simple effects when studying the difference between the times for both groups

|  | **Simple effect between time periods** | **Group** | **T0** | **T1** | **T2** | **T3** | ***P* value (*)** | ***VD.A* (**)** |
| --- | --- | --- | --- | --- | --- | --- | --- | --- |
| **Barthel Index** | T1-T2 | *Delirium* | - | 50 [30-65] | 55 [50-85] | - | <0,001 | 0,246 ^a^ |
|  |  | Non *delirium* | - | 70 [47,5-100] | 90 [55-100] | - | <0,001 | 0,282 ^a^ |
|  | T1-T3 | *Delirium* | - | 50 [30-65] | - | 85 [75-98,8] | <0.001 | 0.134 ^a^ |
|  |  | Non *delirium* | - | 70 [47.5-100] | - | 100 [90-100] | <0.001 | 0.265 ^a^ |
|  | T2-T3 | *Delirium* | - | - | 55 [50-85] | 85 [75-98,8] | <0.001 | 0,176 ^a^ |
|  |  | Non *delirium* | - | - | 90 [55-100] | 100 [90-100] | 0,002 | 0,353 ^c^ |
| **CFS** | T0-T3 | *Delirium* | 3 [3-4] | - | - | 4 [3-4,8] | <0.001 | 0,268 ^a^ |
|  |  | Non *delirium* | 3 [3-4] | - | - | 3 [3-4] | 0.0541 | 0,418 ^c^ |

Data given in median [IQR = interquartile range]; VDA = Vargha and Delaney's A.

* Signs test with Bonferroni adjustment.

** Regarding size effect with VD.A: a large effect (≥0.71 or ≤0.29); b moderate effect (0.64-0.71< or >0.29-0.34); c small effect (between 0.56-0.64 or 0.34-0.44). Values close to 1 or 0 indicate a strong intensity of the mean difference.
